# Effects of Chronological Age, Relative Age, and Maturation Status on Accumulated Training Load and Perceived Exertion in Young Sub-Elite Football Players

**DOI:** 10.3389/fphys.2022.832202

**Published:** 2022-03-31

**Authors:** José Eduardo Teixeira, Ana Ruivo Alves, Ricardo Ferraz, Pedro Forte, Miguel Leal, Joana Ribeiro, António J. Silva, Tiago M. Barbosa, António M. Monteiro

**Affiliations:** ^1^ Research Centre in Sports Sciences, Health and Human Development, Vila Real, Portugal; ^2^ Department of Sports, Exercise and Health Sciences, University of Trás-os-Montes e Alto Douro, Vila Real, Portugal; ^3^ Department of Sport Sciences, Instituto Politécnico de Bragança, Bragança, Portugal; ^4^ Department of Sport Sciences, University of Beira Interior, Covilhã, Portugal; ^5^ Department of Sports, Douro Higher Institute of Educational Sciences, Penafiel, Portugal

**Keywords:** youth, growth, workload, GPS, RPE, heart rate

## Abstract

The aims of this study were 1) to analyze the influence of chronological age, relative age, and biological maturation on accumulated training load and perceived exertion in young sub-elite football players and 2) to understand the interaction effects amongst age grouping, maturation status, and birth quartiles on accumulated training load and perceived exertion in this target population. A 6-week period (18 training sessions and 324 observation cases) concerning 60 young male sub-elite football players grouped into relative age (Q1 to Q4), age group (U15, U17, and U19), and maturation status (Pre-peak height velocity (PHV), Mid-PHV, and Post-PHV) was established. External training load data were collected using 18 Hz global positioning system technology (GPS), heart-rate measures by a 1 Hz short-range telemetry system, and perceived exertion with total quality recovery (TQR) and rating of perceived exertion (RPE). U17 players and U15 players were 2.35 (95% CI: 1.25–4.51) and 1.60 (95% CI: 0.19–4.33) times more likely to pertain to Q1 and Q3, respectively. A negative magnitude for odds ratio was found in all four quartile comparisons within maturation status (95% CI: 6.72–0.64), except for Mid-PHV on Q2 (95% CI: 0.19–4.33). Between- and within-subject analysis reported significant differences in all variables on age group comparison measures (*F* = 0.439 to 26.636, *p* = 0.000 to 0.019, η^2^ = 0.003–0.037), except for dynamic stress load (DSL). Between-subject analysis on maturity status comparison demonstrated significant differences for all training load measures (*F =* 6.593 to 14.424, *p =* 0.000 to 0.037, η^2^ = 0.020–0.092). Interaction effects were found for age group x maturity band x relative age (*Λ* Pillai’s = 0.391, *Λ* Wilk’s = 0.609, *F =* 11.385*, p* = 0.000, η^2^ = 0.391) and maturity band x relative age (*Λ* Pillai’s = 0.252, *Λ* Wilk’s = 0.769, *F =* 0.955*, p* = 0.004, η^2^ = 0.112). Current research has confirmed the effects of chronological age, relative age, and biological maturation on accumulated training load. Perceived exertion does not seem to show any differences concerning age group or maturity status. Evidence should be helpful for professionals to optimize the training process and young football players’ performance.

## Introduction

Monitoring accumulated training load has become a research hot topic in youth football environments (Teixeira et al., 2021a; Miguel et al., 2021). Electronic performance and tracking systems (EPTS) provide affordable and indispensable time-motion technologies for assessing valid training load measures (Linke et al., 2018). The research-practice prominence is due to numerous factors, among which the literature reports practical applicability in the field of strength and conditioning (Cumming et al., 2017), talent identification (Sarmento et al., 2018a), injury prevention (Coppalle et al., 2019; Boullosa et al., 2020), training task design (Coutinho et al., 2017, 2020), and control and performance analysis (Sarmento et al., 2018b; Zurutuza et al., 2017). For several years, literature has reported widely on load terminology such as work rate (O’Donoghue, 2004; Carling et al., 2008), workload (Bowen et al., 2017; Williams et al., 2017; Gabbett et al., 2019), and training load (Impellizzeri et al., 2005; Bourdon et al., 2017; Vanrenterghem et al., 2017). These assumptions are based on a linear perspective where the smallest changes in the system input determine proportional and measurable changes in the output (Vanrenterghem et al., 2017; Impellizzeri et al., 2019). Therefore, the training load concept has been developed by setting up athlete monitoring as a linear system using cumulative effect as a key guidance (Coutts et al., 2017). Cumulative effect is a primary factor to be considered on long-term athletic preparation (Lloyd & Oliver, 2012; Sarmento et al., 2018a).

Typical accumulated training load has already been evaluated in young elite and sub-elite football players (Wrigley et al., 2012; Abade et al., 2014; Coutinho et al., 2015; Teixeira et al., 2021b). An age-related influence was reported for both competitive levels; however, some differences are verified in other independent and conditioned variables, such as playing position and periodization structure. Abade et al. (2014) reported higher total distances covered in under-17 (U17), followed by under-19 (U19), and under-15 (U15) elite football players. Authors also reported lower total and relative body impacts in U15 players. Coutinho et al. (2015) reported a higher total distance covered, body impacts, and high-intensity running in U19 post-match training sessions. Wrigley et al. (2012) reported a higher total weekly training load for under-18 (U18) players. In sub-elite football training, Teixeira et al. (2021b) reported interaction effects between age group, training day, weekly micro-cycle, and playing position for deceleration and between training day, weekly micro-cycle, and age for total covered distance. Also, Teixeira’s study reported minimum effect for playing position on the weekly training load. The weekly accumulated training load varied according to age group, training day, inter-week, and playing position (Wrigley et al., 2012; Abade et al., 2014; Coutinho et al., 2015; Teixeira et al., 2021b). Nonetheless, the playing position seems to be negligible in relation to age and periodization structure (Teixeira et al., 2021b). Recently, a systematic review summarized the studies which have examined external and internal training intensity monitoring; however, the influence of maturity and relative age on accumulated training load and perceived exertion has not been described in any of the available studies (Oliveira et al., 2021).

Relative age and biological maturation were independent and non-modifiable factors that should be considered in player selection, as well as in monitoring and assessment performance in youth football (Cumming et al., 2017; Lovell et al., 2019; Hill et al., 2020). Biological maturation refers to progress towards the adult or mature state defined by status, timing, and tempo (Malina et al., 2015, 2019). According to the maturation-matching assumptions reported by Malina et al. (2019), maturation status, timing, and tempo are distinct concepts: 1) maturation status is the specific maturation stage at the observation time, expressed for instance as skeletal age and stage of pubic hair development; 2) maturation timing is the age at the specific maturational event occurrence, expressed as age at peak height velocity (PHV); and 3) maturation tempo reports the maturation progress in a specific system. Otherwise, relative age refers to a player’s chronological age regarding the competitive cohort and is determined by quartile birth and the competition age-group cohort (Patel et al., 2019, 2020; Hill et al., 2020). Previous studies have demonstrated the influence of relative age, maturation, and anthropometry on physical performance characteristics in elite youth football (Patel et al., 2019, 2020). This evidence seems to be particularly relevant for high-intensity variables, such as sprinting or acceleration (Edwards et al., 2021; Kelly et al., 2021), as well as perceived exertion (Cumming et al., 2018; Hill et al., 2021). Relative age effect (RAE) has been demonstrated within different elite youth football academies (Skorski et al., 2016; Rubajczyk and Rokita, 2018; Hill et al., 2020). Hence, a player selection bias is a consequence of RAE, due to the inter- and intra-variability inherent in biological maturation (Simmons and Paull, 2001; Saavedra-García et al., 2019; Sarmento et al., 2018b). Indeed, differences amongst maturity status and relative age have been identified in previous investigations, along with a considerable variation in timing and rate for physical and biological maturation (Johnson et al., 2017; Towlson et al., 2017). In football, RAE can affect the beginning of a senior career in football with a large over-representation of players born close to the end of the calendar year (Lupo et al., 2019a). Thus, identifying player advances and delays in growth and maturation plays a key role when evaluating player fitness, considering the role of development stages (Cumming, 2018; Ryan et al., 2018).

The analysis of the dependency amongst training load variations and maturational variables has been reported in the literature on elite youth football players (Nobari et al., 2021a, 2021b; Salter et al., 2021). The survey reports the influence of accumulated training load and maturation status in the differences observed across the season (Nobari et al., 2021a). As well known, no studies have included maturational and birth quartile variables to monitor accumulated training load and perceived exertion in sub-elite youth football. Moreover, training load quantification often uses the age-grouping approach instead of bio-banding strategies to compare inter- and intra-individual differences in weekly accumulated training load (Cumming et al., 2017). Previous studies have analyzed the influence of chronological age, relative age, and maturation from standardized physical fitness assessment (Clemente et al., 2019; Nobari et al., 2021a, 2021b; Salter et al., 2021), however this does not include the accumulative outcomes of external and internal training intensity (Oliveira et al., 202; Otte et al., 2019). Upon that, the aims of this study were to 1) to analyze the influence of chronological age, relative age, and biological maturation on accumulated training load and perceived exertion in sub-elite young football players and 2) to understand the interaction effects amongst age grouping, maturation status, and birth quartiles on accumulated training load and perceived exertion in this target population. Based on the relevant literature, we hypothesized that accumulated training load and perceived exertion in young football players will be influenced by advanced growth, relative age, and biological maturation (Clemente et al., 2019; Nobari et al., 2021a, 2021b).

## Methods

### Participants

Participants were sampled from a sub-elite Portuguese youth football academy certified via a zero to four star scale by the Portuguese Football Federation (Santos et al., 2021). A total of 60 male football players was included in this study using an observational, cross-sectional, and convenience sample. Table 1 shows the description baseline characteristics of participants according to age group (i.e., U15, U17, and U19 players) and maturation status (i.e., Pre-, Mid-, and Post-PHV.

**TABLE 1:** Description baseline characteristics of participants.

Group	Age (y)	Relative age	Maturity offset	Height (m)	Weight (kg)	BMI (kg/m^2^)	Sitting height (cm)	PHV (cm)	Experience (y)
U15 (n = 102)	13.28 ± 0.49	0.25 ± 0.17	-0.42 ± 0.76	1.69 ± 0.78	55.67 ± 9.41	19.29 ± 1.99	81.96 ± 5.78	14.18 ± 0.80	4.82 ± 0.90
U17 (n = 99)	15.39 ± 0.51	0.25 ± 0.17	2.02 ± 1.09	1.76 ± 0.48	64.28 ± 6.61	20.68 ± 1.79	92.02 ± 7.61	13.90 ± 1.09	6.64 ± 1.65
U19 (n = 120)	17.29 ± 0.55	0.24 ± 0.20	2.23 ± 1.49	1.76 ± 0.70	68.90 ± 8.39	22.11 ± 1.50	90.73 ± 8.06	14.46 ± 1.87	8.81 ± 1.70
Pre-PHV (n = 52)	13.08 ± 0.39	0.22 ± 0.16	-0.98 ± 0.48	1.68 ± 0.07	56.27 ± 8.63	19.76 ± 1.95	78.08 ± 3.98	14.52 ± 0.66	3.53 ± 0.29
Mid-PHV (n = 65)	14.38 ± 1.66	0.20 ± 0.17	-0.02 ± 0.31	1.73 ± 0.10	60.20 ± 11.73	19.81 ± 2.10	87.54 ± 7.91	13.31 ± 1.83	4.53 ± 1.23
Post-PHV(n = 207)	15.99 ± 1.40	0.27 ± 0.18	2.34 ± 1.17	1.75 ± 0.06	64.75 ± 8.93	21.08 ± 2.06	91.21 ± 7.43	14.40 ± 1.26	9.23 ± 1.36
ALL (n = 324)	15.19 ± 1.75	0.25 ± 0.18	1.33 ± 1.67	1.74 ± 0.08	62.48 ± 10.03	20.61 ± 2.14	88.36 ± 8.51	14.20 ± 1.39	6.76 ± 1.42

PHV, peak height velocity.

All participants were notified about the study’s aims and risks comprised in the research. The study only included players that signed the informed consent, which was conducted according to the ethical standards of the Declaration of Helsinki. The experimental approach was approved and followed by the local Ethical Committee from University of Trás-os-Montes e Alto Douro (3379-5002PA67807).

### Experimental Approach

The weekly training load was continuously monitored in the three age groups during the first month of the 2019–2020 competitive season. The training data corresponded to a 6-week period (18 training sessions and 324 observation cases). The eligibility criteria for individual data sets considered a competitive one-game week schedule and complete full training sessions. The microcycle was comprised of three training sessions per week (∼90 min). The match data were not included in the analysis. The training days were classified as “match day minus format” (MD): MD-3 (Tuesday), MD-2 (Wednesday), and MD-1 (Friday). Training sessions had, on average, 18 players. All age groups trained on an outdoor pitch of official dimensions (FIFA standard; 100 × 70 m). The training sessions were performed on synthetic turf pitches, between 10:00 AM to 08:00 PM and similar environment conditions (14–20°C; relative humidity 52–66%).

The sampled training sessions were categorized according to a specific focus following the discussion with the coach staff. All sampled training sessions started with a standard warm-up with low-intensity running, dynamic stretching for main locomotive lower limb muscles, technical actions, and ball possession. The weekly training overview presented a potential variable between categories, such as different training modes with emphasis in game-based situations, sport-specific skills, and football-specific exercises (Abade et al., 2014; Zurutuza et al., 2017).

### Procedures

Outfield players were monitored using a portable GPS throughout the whole training session (STATSports Apex^®^, Northern Ireland). The GPS device provided raw position velocity and distance at 18 Hz sampling frequencies, including an accelerometer (100 Hz), magnetometer (10 Hz), and gyroscope (100 Hz). Each player used the micro-technology inside a mini pocket of a custom-made vest supplied by the manufacturer, which was placed on the upper back between both scapulae. All devices were activated 30 min before the training data collection to allow an acceptable clear reception of the satellite signal. Respecting the optimal signal to the measurement of human movement, the match data considered eight available satellite signals as the minimum for the observations (Beato et al., 2018). Validity and reliability of global navigation satellite systems (GNSS), such as GPS tracking, have been well established in the literature (Beato et al., 2018; Nikolaidis et al., 2018; Whitehead et al., 2018). Current variables and thresholds should considerer a small error of around 1–2% reported in the 10 Hz STATSports Apex^®^ units (Beato et al., 2018).

### Anthropometry, Relative Age, and Maturity Status

Anthropometric measures were obtained using standard guidelines (Duncan et al., 2019). Players’ height (m), weight (kg), chronological age (years), sitting height (cm), and experience level (years) were collected at each measurement point. Body mass index (BMI) was calculated by dividing the weight by the square of the height (kg/m^2^). Relative age (a.u.) was calculated as the difference between the player’s birthdate and the cut-off date (31 August) divided by the number of days within a year (i.e., 365 days) (Hill et al., 2020). Birth quartiles dates were categorized into birth quartiles (Q) within each age group as: Q1–September to November; Q2–December to February, Q3–March to May, and Q4–June to August (Patel et al., 2019; Hill et al., 2020). Maturity status was based on a predictive Mirwald’s equation (Mirwald et al., 2002) using chronological age, standing height, sitting height, and body mass, as previously established for youth team sports environments (Coutinho et al., 2020; Arede et al., 2021). Sampled players were grouped into three maturity bands based on the predicted adult height (PHV): <88% (Pre-PHV); 88–95% (Mid-PHV), and >95% (Post-PHV) of the predicted adult stature (Cumming et al., 2017). Maturity timing was estimated by z scores: higher than 0.5 (early status); between -0.5 and +0.5 (average maturity timing; this means the players were considered as average in their maturity stages); and below -0.5 (late maturity timing) (Bradley et al., 2019; Arede et al., 2021).

### External Training Load Measures

External training load was obtained through time-motion data: total distance (TD) covered (m), average speed (AvS), maximal running speed (MRS) (ms^−1^), relative high-speed running (rHSR) distance (m), high metabolic load distance (HMLD) (m), sprinting (SPR) distance (m), dynamic stress load (DSL) (a.u.), number of accelerations (ACC), and number of decelerations (DEC). The GPS software provided information only about the locomotor categories above 19.8 km h^−1^: rHSR (19.8–25.1 km h^−1^) and SPR (>25.1 km h^−1^). Sprints were measured by number and average sprint distance (m). HMLD is a metabolic variable defined as the distance, expressed in meters, covered by a player when the metabolic power exceeds 25.5 W kg^−1^. HMLD variables include all high-speed running and accelerations and decelerations above 3 m s^−2^ (Beato et al., 2019; Gómez-Carmona et al., 2020). Both acceleration variables (ACC/DEC) considered the movements in the maximum intensity zone (>3 m s^−2^ and <3 m s^−2^, respectively). DSL was evaluated by a 100 Hz tri-axial accelerometer integrated into the GPS devices by measuring the sum of the accelerations in the three orthogonal axes of movement (X, Y, and Z planes), so as to measure a composite magnitude vector (expressed as G force) (Beato et al., 2019).

The high-intensity activity thresholds were adapted from previous studies (Teixeira et al., 2021a; Miguel et al., 2021). The GPS variables were recorded for each individual player. Individual training data were eliminated from the analysis whenever players left the training before the end of the session due to erroneous data collection, injury, training absence, or early withdrawal (the exclusion criteria resulted in the elimination of 36 observation cases).

### Heart Rate–Based Measures

Heart rate was recorded by 1 Hz short-range telemetry system GARMIM TM HR band (International Inc., Olathe, KS, USA). Maximum heart rate (HR_max_), average heart rate (AvHR), and percentage of HR_max_ (%HR_max_) values were considered for analysis (Branquinho et al., 2020, 2021). Training impulse was obtained by Akubat TRIMP (Akubat et al., 2012, 2014), reporting a team TRIMP, whose equation is based on individual training load from players’ iTRIMP; however, Akubat TRIMP was calculated as: training duration x 0.2053e^3.5179x^. Where e is the Napierian logarithms, 3.5179 is the e exponent, and x is the HR_ratio_ (Akubat et al., 2012). HR_ratio_ is the same in Banisters TRIMP (Teixeira et al., 2021a), HR_max_ was obtained by the Yo intermittent recovery test level 1 (YYIR1) (Bangsbo et al., 2008; Hung et al., 2020).

### Perceived Exertion

Perceived exertion was measured using the 15-point Portuguese Borg Rating of Perceived Exertion 6–20 Scale (Borg RPE 6–20) (Cabral et al., 2020). The sRPE was obtained by multiplying total duration of training sessions for each individual’s RPE score (sRPE = RPE × session duration) following a scale from 6 to 20 (Haddad et al., 2017). To monitor recovery, each player was asked to report the TQR score on a scale from 6 to 20. This scale was proposed by Kenttä and Hassmén (1998) to measure athletes’ recovery perceptions. Previous research included the TQR score examining perceived stress and fatigue in youth football (Brink et al., 2010; Clemente et al., 2019; Teixeira et al., 2021b). RPE and TQR were collected individually at approximately 30 min after and before each training session, respectively. Players were preemptively familiarized with the procedures, and perceived data were collected using a Microsoft Excel^®^ spreadsheet (Microsoft Corporation, U.S.).

### Statistical Analysis

Robust estimates of 95% confidence interval (CI) and heteroscedasticity were calculated by a bootstrapping technique (randomly 1,000 bootstrap samples) (Beato and Drust, 2021; Maughan et al., 2021). Birth quartile distribution according to group and maturation band were calculated by counts (n), frequencies (%), and odds ratio (OR) (Patel et al., 2019; Hill et al., 2020). Z score was computed to compare accumulated training load measures and perceived measures (Gallo et al., 2016; Cumming et al., 2017). A one-way analysis of variance (ANOVA) was used to identify differences between age groups and maturation bands. A repeated-measures multivariate analysis of variance (MANOVA) was applied to analyze within-subject changes and interaction effects (age group x maturity band x relative age) (Charness et al., 2012; Barbosa et al., 2016). The sample size was calculated with G*Power, Version 3.1.5.1 (Institut für Experimentelle Psychologie, Düsseldorf, Germany), using an effect size ß of 0.4, an α of 0.05, and a power of 0.8 (1–ß) (Abade et al., 2014). For ANOVA repeated-measures within-between interaction, the total sample size computed was 15 subjects. For MANOVA repeated-measures within-between interaction, the total sample size computed was 91. The number of groups and measures considered were 3 and 17, respectively. When a significant difference occurred, Bonferroni post-hoc tests were used to identify localized effects. Games–Howell post-hoc tests were applied if variances were not homogeneous. The effect size eta square (η^2^) was computed and interpreted as: 1) without effect if 0 < η^2^ ≤ 0.04; 2) minimum if 0.04 < η^2^ ≤ 0.25; 3) moderate if 0.25 < η^2^ ≤ 0.64; and 4) strong if η^2^ > 0.64 (Ferguson, 2009). Standardized effect sizes (ES) were calculated with Cohen’s *d* for pairwise comparison, classified as: 0–0.2, trivial; 0–0.6, small; 0.6–1.2, moderate; 1.2–2.0, large; 2.0–4.0 very large effect, and >4 nearly perfect (Hopkins et al., 2009; Barbosa et al., 2018). The intraclass correlation coefficient (ICC) from a two-way random effects model was computed for single (ICC = 0.04, 95% CI: 0.02-0.06) and average measures (ICC = 0.04, 95% CI: 0.25-0.46) with α Cronbach’s coefficient (*α* = 0.36) (Koo and Li, 2016). Statistical significance was set at *p* < 0.05 and data are presented as the mean ± SD. All statistical analyses were conducted using IBM SPSS Statistics for Windows, Version 27.0 (Armonk, NY: IBM Corp) and JASP software (JASP Team, 2019; jasp-stats.org). Data visualization was produced by Graph Pad Prism (GraphPad Software, CA, USA).

## Results

### Baseline Characteristics, Accumulated Training Load, and Perceived Exertion According to Age Group, Maturity Band, and Relative Age (z Score)

**FIGURE 1:**
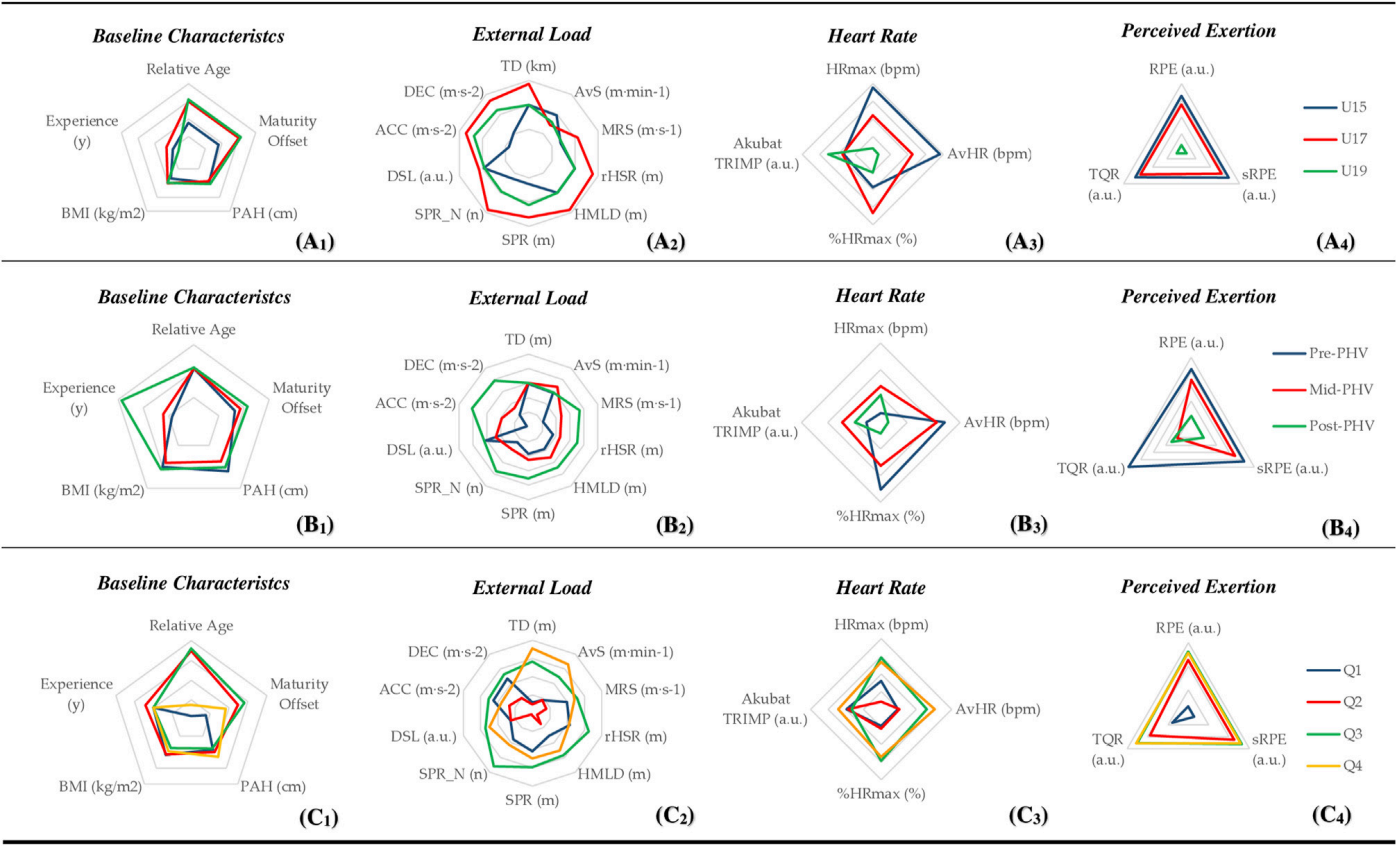
Baseline characteristics accumulated training load and perceived exertion according to age - grouping **(A1-A4)** maturity band **(B1-B4)** and relative age **(C1-C4)** using z score. Abbreviations: ACC - acceleration; AvHR - average heart rate. AvS - average speed; DEC - deceleration; HMLD - high metabolic load distance; HRmax - maximal heart rate; MRS - maximum running speed; n - number of events; RPE - ratings of perceived exertion; SPR - average sprint distance; SPR_N - number of sprints; sRPE - session ratings of perceived exertion; TD - total distance; TQR - total quality recovery; TRIMP - training impulse; U - Under; DSL - Dynamic stress load; PHV - peak height velocity.

Figure 1 shows the baseline characteristics, accumulated training load, and perceived exertion according to age group, maturity band, and relative age using z scores.

Table 2 presents the OR, frequency, and distribution of birth quartiles relating to age group, maturity band, and overall population. U17 players were 2.35 (95% CI: 1.25–4.51) times more likely to fall into Q1 whereas there was the slightest chance to be in Q2 (95% CI: 2.38–-0.60) and Q3 (95% CI: 2.38–-0.60). U15 players were 1.60 (95% CI: 0.19–4.33) times more likely to be in Q3. A negative magnitude for OR was found in all quartile comparisons within maturation status (95% CI: 6.72–0.64), except for Mid-PHV on Q2 (95% CI: 0.19–4.33). Only Pre-PHV did not show statistically significant differences (*p* = 0.054).

**TABLE 2:** Odds ratio, frequency, and distribution of birth quartiles relating to age group, maturity band, and overall population.

Variables	Q1 *n* (%)	Q2 *n* (%)	Q3 *n* (%)	Q4 *n* (%)	Q1 vs. Q4^a^ OR (95%CI)	Q2 vs. Q4* OR (95%CI)	Q3 vs. Q4* OR (95%CI)	*p value*
**Age group**								
U15 (n = 102)	10 (13.69)	27 (22.98)	31 (36.69)	34 (36.96)	1.87 (−0.35–4.94)	−0.87 (−2.64–1.31)	1.60 (0.19–4.33)	0.001
U17 (n = 99)	18 (22.31)	11 (15.07)	37 (43.02)	33 (35.87)	2.35 (1.25–4.51)	−1.43 (−2.38–−0.60)	0.46 (−0.31–1.23)	0.001
U19 (n = 120)	45 (61.64)	35 (47.95)	18 (20.93)	25 (27.17)	—	—	—	—
**Maturation band**								
Pre-PHV (n = 52)	5 (9.62)	22 (42.31)	5 (9.62)	20 (38.46)	−3.57 (−6.72–−2.12)	0.62 (−1.60–2.60)	−2.68 (−6.02–−1.12)	0.054
Mid-PHV (n = 65)	24 (36.92)	5 (7.69)	21 (32.31)	15 (23.08)	−1.34 (−2.25–−0.51)	−0.61 (−2.59–1.07)	−0.82 (−3.14–0.64)	0.003
Post-PHV (n = 207)	44 (21.26)	46 (22.22)	60 (28.99)	57 (27.54)	—	—	—	—

* Reference group; significant differences are verified as: (a) Q1 vs. Q4; (b) Q2 vs. Q4; (c) Q3 vs. Q4. *Abbreviations*: CI, confidence interval; PHV, peak height velocity; OR, odds ratio; Q–quartile; U–under.

### Effect of Age Group, Group Maturation Status, and Relative Age on Accumulated Training Load and Perceived Exertion

Inferential analysis is displayed in Table 3 and Table 4, reporting between- and within-subject differences for age group and maturity band. Between-subject analysis reported significant differences in all variables on age group comparison (*F =* 0.439 to 26.636, *p =* 0.000 to 0.019, η^2^ = 0.003–0.037), except in DSL (*F =* 0.439, *p =* 0.645, η^2^ = 0.003). All external training load measures had a higher magnitude on U17 players, as well HRmax, Akubat TRIMP, and TQR. AvHR, %HRmax, RPE, and sRPE were greater in U15 players. Furthermore, within-subject analysis also showed no significant differences in DSL (*F =* 0.512, *p =* 0.600, η^2^ = 0.003). Also, differences without statistical significance were found for HMLD (*F =* 5.599, *p =* 0.124, η^2^ = 0.004), HRmax (*F =* 2.103, *p =* 0.124, η^2^ = 0.014), and perceived exertion (*F =* 0.103 to 0.853, *p =* 0.427 to 0.94, η^2^ = 0.000–0.006).

**TABLE 3:** Mean external training load, heart rate-based measures, and perceived exertion for each age group examined.

Variables	*Age group*			*Between-subject*			*Within-subject*			*Post-hoc*
	U15 (n = 20)	U17 (n = 20)	U19 (n = 20)	*F*	*p*	*η* ^*2*^	*F*	*p*	*η* ^*2*^	
**External load**										
TD (m)	5,316.18 ± 1,354.45	6,021.45 ± 1,675.64	4,750.43 ± 1,593.46	18.465	0.000	0.103	7.442	0.001	0.047	a,b,c
AvS (m·min^−1^)	49.96 ± 16.35	56.84 ± 34.51	45.83 ± 15.60	6.192	0.002	0.037	4.550	0.011	0.030	a
MRS (m·s^−1^)	6.58 ± 0.82	7.94 ± 3.12	7.43 ± 1.15	13.014	0.000	0.075	5.125	0.013	0.033	a,b
rHSR (m)	53.23 ± 58.34	166.06 ± 458.95	72.41 ± 65.95	5.525	0.004	0.033	4.398	0.007	0.013	a,c
HMLD (m)	489.11 ± 228.44	730.56 ± 483.38	524.90 ± 291.37	14.395	0.000	0.082	5.599	0.124	0.004	a,c
SPR (m)	28.13 ± 41.66	130.42 ± 462.56	40.16 ± 50.43	4.773	0.009	0.029	4.032	0.019	0.026	a,c
SPR_N (n)	1.85 ± 2.46	4.83 ± 4.81	3.12 ± 2.92	18.363	0.000	0.103	4.335	0.014	0.028	a,b,c
DSL (a.u.)	247.21 ± 135.86	261.28 ± 121.73	245.19 ± 144.87	0.439	0.645	0.003	0.512	0.600	0.003	-
ACC (m·s^−2^)	33.62 ± 18.80	53.76 ± 20.62	49.90 ± 20.19	26.636	0.000	0.156	11.43	0.000	0.071	a,b
DEC (m·s^−2^)	30.27 ± 19.77	49.77 ± 25.08	44.01 ± 22.53	20.103	0.000	0.111	7.378	0.001	0.047	a,b
**Heart rate**										
HRmax	185.69 ± 9.96	188.64 ± 9.09	184.00 ± 10.54	3.057	0.048	0.019	2.103	0.124	0.014	b
AvHR	140.00 ± 9.71	136.53 ± 10.91	132.15 ± 11.52	6.024	0.003	0.036	4.996	0.007	0.032	b,c
%HRmax	75.71 ± 5.26	73.79 ± 5.93	71.65 ± 204.88	14.963	0.000	0.085	6.634	0.002	0.042	b,c
Akubat TRIMP	91.90 ± 34.18	94.03 ± 31.78	81.56 ± 29.13	5.069	0.007	0.082	3.983	0.020	0.026	c
**Perceived exertion**										
RPE (a.u.)	13.73 ± 1.91	13.51 ± 1.76	12.45 ± 2.50	11.964	0.000	0.069	0.853	0.427	0.006	a,c
sRPE (a.u.)	2056.51 ± 171.87	1835.40 ± 158.71	2497.99 ± 224.69	11.964	0.000	0.069	0.853	0.427	0.006	a,c
TQR (a.u.)	16.38 ± 1.92	16.24 ± 1.81	15.21 ± 2.16	11.923	0.000	0.103	0.062	0.940	0.000	a,c

Significant differences are verified as: (a) U15 vs. U17; (b) U15 vs. U19; (c) U17 vs. U19. *Abbreviations*: ACC, acceleration; AvHR, average heart rate. AvS–average speed; DEC–deceleration; HMLD, high metabolic load distance; HRmax, maximal heart rate; MRS, maximum running speed; n–number of events; RPE, ratings of perceived exertion; SPR, average sprint distance; SPR_N - number of sprints; sRPE, session ratings of perceived exertion; TD, total distance; TQR, total quality recovery; TRIMP, training impulse; U–under.

**TABLE 4:** Mean external training load, heart rate-based measures, and perceived exertion for each age group examined.

Variables	*Maturity band*			*Between-subject*			*Within-subject*			*Post-hoc*
	*Pre-PHV (n = 52)*	*Mid-PHV (n = 65)*	*Post-PHV (n = 207)*	*F*	*p*	*η* ^*2*^	*F*	*p*	*η* ^*2*^	
**External load**										
TD (m)	5,302.62 ± 1,444.91	5,323.92 ± 1,559.22	5,318.28 ± 1702.01	0.003	0.997	0.000	7.442	0.001	0.047	-
AvS (m·min^−1^)	49.923 ± 16.35	52.39 ± 25.91	50.04 ± 24.27	0.260	0.771	0.002	4.550	0.011	0.030	-
MRS (m·s^−1^)	6.55 ± 0.93	7.08 ± 1.64	7.59 ± 2.22	6.593	0.002	0.039	5.125	0.013	0.033	b
rHSR (m)	57.65 ± 65.68	65.85 ± 69.67	84.85 ± 82.19	3.400	0.035	0.037	4.398	0.007	0.013	-
HMLD (m)	492.25 ± 244.24	533.99 ± 271.52	582.30 ± 304.15	2.301	0.102	0.014	5.599	0.124	0.004	-
SPR (m)	49.02 ± 17.46	52.39 ± 25.91	50.04 ± 25.91	3.335	0.037	0.020	4.032	0.019	0.026	-
SPR_N (n)	6.55 ± 0.93	7.07 ± 1.64	7.59 ± 22.22	7.268	0.001	0.043	4.335	0.014	0.028	b,c
DSL (a.u.)	253.44 ± 133.61	233.31 ± 135.73	255.54 ± 135.43	0.681	0.507	0.004	0.512	0.600	0.003	b,c
ACC (m·s^−2^)	33.48 ± 18.71	41.29 ± 21.98	50.55 ± 20.66	16.293	0.000	0.092	11.43	0.000	0.071	b,c
DEC (m·s^−2^)	32.04 ± 20.97	34.82 ± 21.51	45.88 ± 24.12	10.773	0.000	0.063	7.378	0.001	0.047	b,c
**Heart rate**										
HRmax	184.63 ± 10.64	186.69 ± 8.34	186.05 ± 10.46	6.024	0.003	0.036	0.191	0.826	0.001	b,c
AvHR	139.17 ± 10.61	138.48 ± 9.45	134.36 ± 11.65	14.963	0.000	0.085	0.642	0.527	0.004	-
%HRmax	75.40 ± 5.70	74.29 ± 5.44	72.81 ± 6.30	14.424	0.000	0.082	0.969	0.381	0.006	b,c
Akubat TRIMP	88.67 ± 36.80	92.37 ± 27.69	87.44 ± 32.03	1.105	0.333	0.007	1.277	0.280	0.008	b,c
**Perceived exertion**										
RPE (a.u.)	13.65 ± 1.83	13.49 ± 2.07	12.95 ± 2.28	3.057	0.048	0.019	0.211	0.810	0.001	b,c
sRPE (a.u.)	1,228.85 ± 164.21	1,214.31 ± 186.30	1,165.65 ± 204.88	3.057	0.048	0.019	0.211	0.810	0.001	b,c
TQR (a.u.)	16.50 ± 1.79	15.71 ± 2.23	15.80 ± 2.03	2.789	0.063	0.017	0.300	0.741	0.002	-

Significant differences are verified as: (a) U15 vs. U17; (b) U15 vs. U19; (c) U17 vs. U19. *Abbreviations*: ACC, acceleration; AvHR, average heart rate. AvS–average speed; DEC–deceleration; HMLD, high metabolic load distance; HRmax, maximal heart rate; MRS, maximum running speed; n–number of events; RPE, ratings of perceived exertion; SPR, average sprint distance; SPR_N - number of sprints; sRPE, session ratings of perceived exertion; TD, total distance; TQR, total quality recovery; TRIMP, training impulse; U–under.

Between-subject analysis on maturity band comparison demonstrated significant differences for external measures, specifically MRS (*F =* 6.593, *p =* 0.002, η^2^ = 0.039), rHSR (*F =* 3.400, *p =* 0.035, *η* ^*2*^ = 0.037), SPR (*F =* 3.335, *p =* 0.037, η^2^ = 0.020), SPR_N (*F =* 7.268, *p =* 0.001, η^2^ = 0.043), ACC (*F =* 16.293, *p =* 0.000, η^2^ = 0.092), and DEC (*F =* 10.773, *p =* 0.000, *η* ^*2*^ = 0.063). Post-PHV players covered all of these variables with statistical significance, except SPR which showed higher values in the Pre-PHV players. Heart rate measures exhibited significant differences for HRmax (*F =* 6.024, *p =* 0.002, η^2^ = 0.039), AvHR (*F =* 14.963, *p =* 0.000, η^2^ = 0.085), and %HRmax (*F =* 14.424, *p =* 0.000, η^2^ = 0.082). Pre-PHV players had higher AvHR and %HR_max_ compared to the Mid-PHV band. Perceived exertion was higher in Pre-PHV players, without differences, though.

**FIGURE 2:**
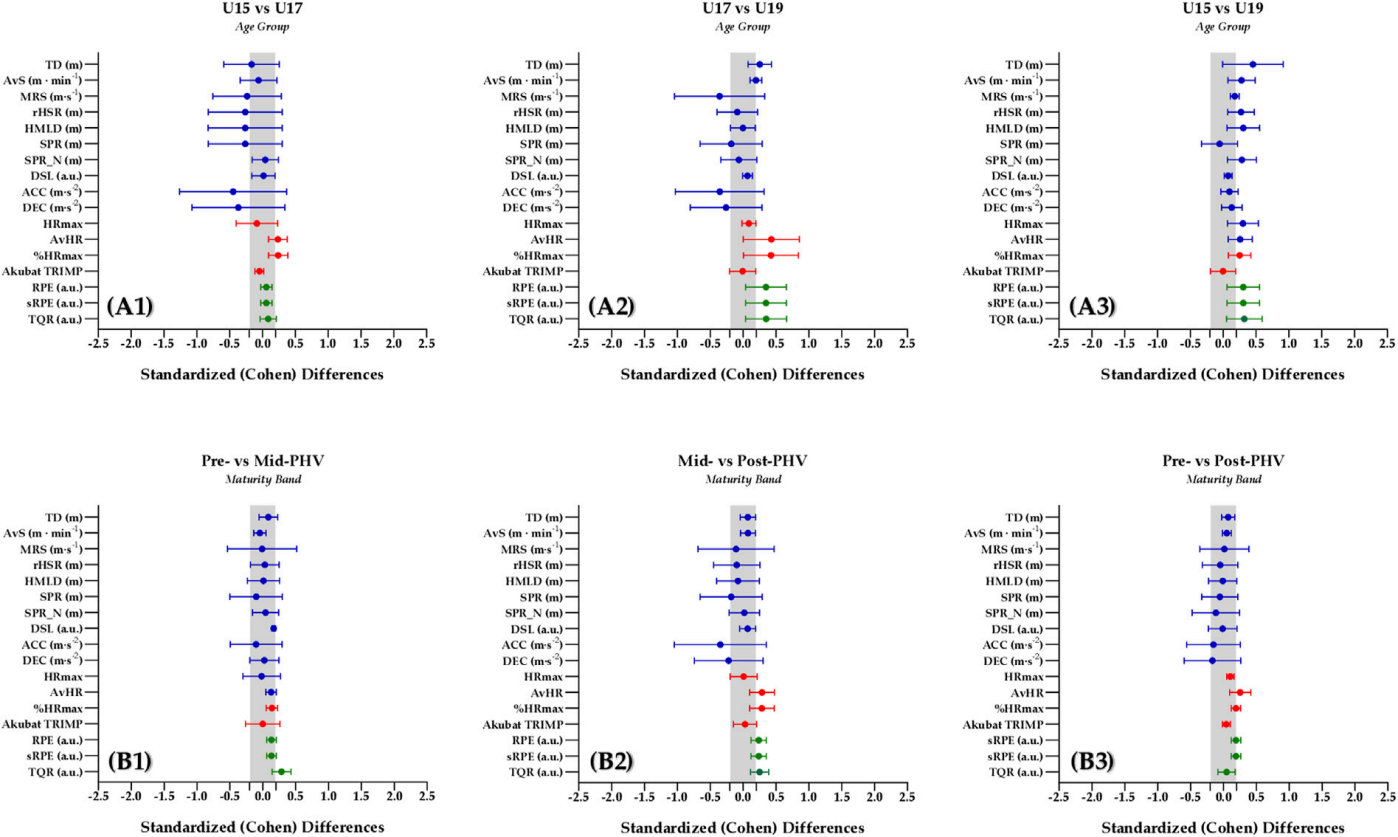
Standardized (Cohen) difference for external training load, hear rate - based measures and perceived exertion according to age group **(A1-A3)** and maturation bands **(B1-B3)** post - hoc comparisons **(A1)** Pre-vs Mid-PHV; **(A2)** Pre-vs Post-PHV; **(A3)** Pre-vs Mid-PHV; **(B1)** Pre-va Mid-PHV; **(B2)** Pre-vs Post-PHV. Significant differences are verified as: **(A)** U15 vs. U17; **(B)** U15 vs. U19; **(C)** U17 vs. U19. Abbreviations: ACC - acceleration; AvHR - average heart rate. AvS - average speed; DEC - deceleration; HMLD - high metabolic load distance; HRmax - maximal heart rate; MRS - maximum running speed; n - number of events; PHV - peak height velocity; RPE - ratings of perceived exertion; SPR - average sprint distance; SPR_N - number of sprints; sRPE - session ratings of perceived exertion; TD - total distance; TQR - total quality recovery; TRIMP - training impulse; U - Under; DSL - dynamic stress load; PHV - peak height velocity.

The pairwise comparison was analyzed according to age groups (i.e., U15 vs. U17, U17 vs. U19, and U15 vs. U19) and maturity band (i.e., Pre-vs. Mid-PHV, Mid-vs. Post-PHV, and Pre-vs. Post-PHV), reporting the following ES for each variable (negative to large effects): TD (*d* = −0.461–0.779), AvS (*d* = −0.05–0.427), MRS (*d* = −0.602–0.225), rHSR (*d* = −0.662–0.483), HMLD (*d* = −0.600 to −0.249), SPR (*d* =), SPR_N (*d* = -0.516–0.119), DSL (*d* = -0.164–0.441), ACC (*d* = -1.022–0.189), DEC (*d* = -1.022–0.189), HRmax (*d* = -0.308–0.467), AvHR (*d* = 0.070–0.731), %HRmax (*d* = 0.2–0.719), Akubat TRIMP (*d* = -1.86–0.087), RPE (*d* = 0.082–0.568), sRPE (*d* = 0.082–0.568), and TQR (*d* = -0.045–0.571). Previous standardized (Cohen) differences are presented in Figure 2 according to age group and maturity bands for external training load, hear rate-based measures, and perceived exertion.

### Interaction Effects Amongst Age Group, Maturation Status, and Relative Age on Accumulated Training Load and Perceived Exertion

Interaction effects were found for age group x maturity band x relative age (*Λ* Pillai’s = 0.391, *Λ* Wilk’s = 0.609, *F =* 11.385*, p* = 0.000, η^2^ = 0.391) and maturity band x relative age (*Λ* Pillai’s = 0.252, *Λ* Wilk’s = 0.769, *F =* 0.955*, p* = 0.004, η^2^ = 0.112). No isolated interaction was found for age group x maturity band (*Λ* Pillai’s = 0.122, *Λ* Wilk’s = 0.881, *F =* 1.159*, p* = 0.066, η^2^ = 0.083) and age group x relative age (*Λ* Pillai’s = 0.327, *Λ* Wilk’s = 0.710, *F =* 1.261*, p* = 0.064, η^2^ = 0.065).

## Discussion

The aims of this study were to analyze the influence of chronological age, relative age, and biological maturation on accumulated training load and perceived exertion in young sub-elite football players. Also, we intended to understand the interaction effects amongst age grouping, maturation status, and birth quartiles on accumulated training load and perceived exertion in this target population.

### Effect of Age Group, Group Maturation Status, and Relative Age on Accumulated Training Load and Perceived Exertion

Current research has confirmed an RAE on accumulated training load and perceived exertion when using an annual age-grouping strategy. U17 players and U15 players were 2.35 (95% CI: 1.25–4.51) and 1.60 (95% CI: 0.19–4.33) times more likely to pertain to Q1 and Q3. The obtained results were congruent with the hypothesis raised, being able to assume that the relative age and biological maturation would have an influence on the accumulated training load and perceived exertion in young football players. Previous research has reported a selection bias with an age-related increase with a maturity dependence over U12 age groups (Hill et al., 2020). Indeed, RAE increased linearly with relative age differences depending on category, skill level, and sport context (Cobley et al., 2009). However, previous studies have pointed out that baseline characteristics did not differ by birth quartile except for age and PHV (Figueiredo et al., 2019). Inconsistencies have been reported when seeking to understand the RAE in physical demands (Hill et al., 2020; Patel et al., 2020; Figueiredo et al., 2021). Patel et al. (2020) reported that somatic maturity and anthropometric and physical performance characteristics distinguished retained or dropout individuals in an age group-dependent manner as opposed to birth quartile. The present study found a negative magnitude for all quartile comparisons within maturation status (95% CI: 6.72–0.64), except for Mid-PHV on Q2 (95% CI: 0.19–4.33). In line with these assumptions, the literature recommends strategies to reduce selection biases and better identify, retain, and develop football players (Hill et al., 2020). Applying a bio-banding process could also bring potential benefits for strength and conditioning of youth athletes (Malina et al., 2015, 2019; Cumming et al., 2017). For these reasons, the present study also sought to establish the differences between age groups and biological band cut-offs on accumulated training load and perceived exertion.

Between- and within-subject analysis reported significant differences in all variables on age group comparison, except in DSL. Also, within-subject analysis also showed no significant differences for HMLD, HRmax, and perceived exertion. Several studies have demonstrated an age-related influence on physical performance characteristics in young elite football players (Rubajczyk and Rokita, 2018; Patel et al., 2019; Edwards et al., 2021). However, both match running and training load seem to be more influenced by biological maturation and anthropometry than by individual chronological age (Nobari et al., 2021a; Salter et al., 2021). The results of the present research seem to corroborate this evidence since the between-subject analysis on maturity band comparison demonstrated significant differences for external measures. Post-PHV players presented high demands in all external training loads, except pre-PHV players. In contrast, Nobari et al. (2021a) achieved no significant differences in accumulated training load and maturation status in U16 players. The authors controlled seasonal phases unlike the current study. It is therefore difficult to generalize RPE outcomes, due to the wide variation according to different training settings, players, and coaches’ strategies (Lupo et al., 2019b). Thus, futures research should also include the influence of seasonal variation on the accumulated training load as has been reported previously for sub-elite youth football (Teixeira et al., 2021a; 2021b). Indeed, weekly accumulated training load varied according to age group, training day, inter-week, and playing position (Teixeira et al., 2021b). An intra-individual variation was also reported in youth-perceived exertion in different intensity training sessions (Lupo et al., 2017). The current research demonstrated differences in the internal training load and perceived exertion amongst maturation bands. Pre-PHV players had a higher AvHR and %HRmax compared to the Mid-PHV band. Perceived exertion was higher in Pre-PHV players, without differences, though. Previous research has established that perceived exertion seems to be better explained by variables associated with trainability, maturation, and stage of development than by training conditions or demands (Malina et al., 2019). Perceived exertion in young football players may also be influenced by psycho-physiological determinants, such as self-perception of competence and practice experience (Ferraz et al., 2018a, 2020; Branquinho et al., 2021). Also, the influence of wellness status must also be considered on accumulated training load and perceived exertion (Clemente et al., 2017, 2020).

### Interaction Effects Amongst Age Group, Maturation Status, and Relative Age on Accumulated Training Load and Perceived Exertion

Multivariate interaction effects amongst conditioned factor and accumulated training load have been previously reported in elite and sub-elite football environments (Teixeira et al., 2021b; Maughan et al., 2021). Current findings displayed an interaction effect between age group x maturity band x relative age and maturity band x relative age. This study adds new practical insights as previous research had not considered maturational variables in training load variability (Maughan et al., 2021; Teixeira et al., 2021a, 2021b). Maughan et al. (2021) described the main effects amongst playing position and stage of season for training and match load. Teixeira et al. (2021b) observed interaction effect for TD between inter-day, inter-week, and age, as well as amongst the inter-day, inter-week, age group, and playing position for DEC. Otherwise, the playing position effect on physical demands seems to be distinguished in elite and sub-elite contexts (Teixeira et al., 2021b; Maughan et al., 2021). The literature reported a minimum effect on the weekly training in sub-elite youth football training (Teixeira et al., 2021b). Negative to large effects were reported in the current research, however the magnitude of effects should be interpreted differently depending on the targeting group as well as the training setting (Flanagan, 2013). Albeit, seasonal loading variation seems to be influenced by seasonal factors as well as others conditioned and independent factors (i.e., weekly microcycle, player’s starting status, training mode, and contextual variables) (Teixeira et al., 2021a). Within-athlete variability should also be considered to evaluate training load and perceived exertion across the competitive season (Malcata and Hopkins, 2014; Younesi et al., 2021). The classic concept of training cycles (Issurin, 2010) tends not to exist in the coaching context of team sports such as football, particularly during competitive periods (Brito et al., 2016; Clemente et al., 2020; Nobari et al., 2021a). Here, there is a regular methodological pattern (i.e., weekly pattern) as presented in elite young players (Wrigley et al., 2012; Abade et al., 2014; Coutinho et al., 2015). Recently, a systematic review clarified a high load variation in a weekly microcycle and a limited variation across a competitive season in elite and sub-elite football (Teixeira et al., 2021a; 2021b). The current research adds evidence for an interactive influence of chronological age, relative age, and maturation in weekly training load patterns at sub-elite football environments. Coaches should be advised to design and prescribe internal and external training intensity, adjusting stimulus into growth and maturity status of the youth football players.

### Limitations and Future Perspectives

The current study has some limitations, which affect how the results should be interpreted: 1) match data were not included in the present data analysis, and periodization structure considers the whole training session. Indeed, match running performance should also be included as well as the contextual factor influence (Gonçalves et al., 2019; Teixeira et al., 2021c); 2) technical factors (i.e., running with or without the ball) (Yi et al., 2020a; 2020b), tactical key indicators (i.e., possession strategies) (Bradley et al., 2014), and collective behavior must be considered for a more integrative and ecological analysis (Bradley and Ade, 2018; Carling, 2013; Ferraz, et al., 2018b; Folgado et al., 2018; Gonçalves et al., 2018); 3) the methodological bias evidenced for the different formulas to estimate maturity state should be considered when interpreting current findings (Malina et al., 2015; Cumming, 2018); 4) cumulative effects of pre-match training were not controlled in this research (Branquinho et al., 2021; Trecroci, et al., 2020a, 2020b); and 5) current training data reflect only one sub-elite football academy and hence cannot be extended to other contexts. Hence, more analyses are required for this purpose, with a wider follow-up. Future research should also consider the relationship of accumulated training load, such as congested fixture, players’ starting status, and competitive level (Teixeira et al., 2021a; 2021b). It would be likewise pertinent to study female football players to develop the generalizability of the achieved results (Nobari et al., 2021c). The comparison between bio-banding and age-grouping should also be analyzed using a quasi-experimental methodology and not just an observational prospective (Arede et al., 2021). Individualized reference values for internal and external training intensity in sub-elite training insights is another key point to explore (Boullosa et al., 2020; Rago et al., 2020; Oliveira et al., 2021). Also, resultant composition equations should be developed to extract meaning in the emergence of new source information (Rojas-Valverde et al., 2019, 2020).

## Conclusion

The current research has confirmed an interaction effect amongst chronological age, relative age, and maturation on accumulated training load, however perceived exertion does not seem to differ either in age group and maturity status. Also, the within-between interaction showed significant differences in all variables on age group and maturation status comparison. This study provides new useful insights to prescribe and control training load in young sub-elite football players.

## Data Availability

The raw data supporting the conclusions of this article will be made available by the authors, without undue reservation.

## References

[B1] Abade E. A. Gonçalves B. V. Leite N. M. Sampaio J. E. (2014). Time-Motion and Physiological Profile of Football Training Sessions Performed by Under-15, Under-17, and Under-19 Elite Portuguese Players. Int. J. Sports Physiol. Perform. 9, 463–470. 10.1123/ijspp.2013-0120 23920425

[B2] Akubat I. Barrett S. Abt G. (2014). Integrating the Internal and External Training Loads in Soccer. Int. J. Sport Physiol. Perform. 9, 457–462. 10.1123/IJSPP.2012-0347 23475154

[B3] Akubat I. Patel E. Barrett S. Abt G. (2012). Methods of Monitoring the Training and Match Load and Their Relationship to Changes in Fitness in Professional Youth Soccer Players. J. Sports Sci. 30, 1473–1480. 10.1080/02640414.2012.712711 22857397

[B4] Arede J. Cumming S. Johnson D. Leite N. (2021). The Effects of Maturity Matched and Un-matched Opposition on Physical Performance and Spatial Exploration Behavior during Youth Basketball Matches. PLoS ONE 16 (4), e0249739. 10.1371/journal.pone.0249739 33831106 PMC8031392

[B5] Bangsbo J. Iaia F. M. Krustrup P. (2008). The Yo-Yo Intermittent Recovery Test. Sports Med. 38, 37–51. 10.2165/00007256-200838010-00004 18081366

[B6] Barbosa T. M. Goh W. X. Morais J. E. Costa M. J. Pendergast D. (2016). Comparison of Classical Kinematics, Entropy, and Fractal Properties as Measures of Complexity of the Motor System in Swimming. Front. Psychol. 7, 1566. 10.3389/fpsyg.2016.01566 27774083 PMC5053984

[B7] Barbosa T. M. Ramos R. Silva A. J. Marinho D. A. (2018). Assessment of Passive Drag in Swimming by Numerical Simulation and Analytical Procedure. J. Sports Sci. 36, 492–498. 10.1080/02640414.2017.1321774 28453398

[B8] Beato M. Coratella G. Stiff A. Iacono A. D. (2018). The Validity and Between-Unit Variability of GNSS Units (STATSports apex 10 and 18 Hz) for Measuring Distance and Peak Speed in Team Sports. Front. Physiol. 9, 1–8. 10.3389/fphys.2018.01288 30298015 PMC6161633

[B9] Beato M. De Keijzer K. L. Carty B. Connor M. (2019). Monitoring Fatigue during Intermittent Exercise with Accelerometer-Derived Metrics. Front. Physiol. 10, 780. 10.3389/fphys.2019.00780 31293447 PMC6606691

[B10] Beato M. Drust B. (2021). Acceleration Intensity Is an Important Contributor to the External and Internal Training Load Demands of Repeated Sprint Exercises in Soccer Players. Res. Sports Med. 29, 67–76. 10.1080/15438627.2020.1743993 32200649

[B11] Boullosa D. Casado A. Claudino J. G. Jiménez-Reyes P. Ravé G. Castaño-Zambudio A. (2020). Do you Play or Do You Train? Insights from Individual Sports for Training Load and Injury Risk Management in Team Sports Based on Individualization. Front. Physiol. 11, 995. 10.3389/fphys.2020.00995 32973548 PMC7472986

[B12] Bourdon P. C. Cardinale M. Murray A. Gastin P. Kellmann M. Varley M. C. (2017). Monitoring Athlete Training Loads: Consensus Statement. Int. J. Sports Physiol. Perform. 12, S2–S161. 10.1123/IJSPP.2017-0208 28463642

[B13] Bowen L. Gross A. S. Gimpel M. Li F.-X. (2017). Accumulated Workloads and the Acute:chronic Workload Ratio Relate to Injury Risk in Elite Youth Football Players. Br. J. Sports Med. 51, 452–459. 10.1136/bjsports-2015-095820 27450360 PMC5460663

[B14] Bradley B. Johnson D. Hill M. McGee D. Kana-ah A. Sharpin C. (2019). Bio-banding in Academy Football: Player's Perceptions of a Maturity Matched Tournament. Ann. Hum. Biol. 46, 400–408. 10.1080/03014460.2019.1640284 31288575

[B15] Bradley P. S. Ade J. D. (2018). Are Current Physical Match Performance Metrics in Elite Soccer Fit for Purpose or Is the Adoption of an Integrated Approach Needed? Int. J. Sports Physiol. Perform. 13, 656–664. 10.1123/ijspp.2017-0433 29345547

[B16] Bradley P. S. Lago-Peñas C. Rey E. Sampaio J. (2014). The Influence of Situational Variables on ball Possession in the English Premier League. J. Sports Sci. 32, 1867–1873. 10.1080/02640414.2014.887850 24786661

[B17] Branquinho L. Ferraz R. Travassos B. C. Marques M. (2020). Comparison between Continuous and Fractionated Game Format on Internal and External Load in Small-Sided Games in Soccer. Ijerph 17, 405. 10.3390/ijerph17020405 PMC7014000 31936244

[B18] Branquinho L. Ferraz R. Travassos B. Marinho D. A. Marques M. C. (2021). Effects of Different Recovery Times on Internal and External Load during Small-Sided Games in Soccer. Sports Health 13, 194173812199546. 10.1177/1941738121995469 PMC8645317 33622118

[B19] Brink M. S. Nederhof E. Visscher C. Schmikli S. L. Lemmink K. A. P. M. (2010). Monitoring Load, Recovery, and Performance in Young Elite Soccer Players. J. Strength Cond. Res. 24, 597–603. 10.1519/JSC.0b013e3181c4d38b 20145570

[B20] Brito J. Hertzog M. Nassis G. P. (2016). Do match-related Contextual Variables Influence Training Load in Highly Trained Soccer Players? J. Strength Cond. Res. 30, 393–399. 10.1519/JSC.0000000000001113 26244827

[B21] Cabral L. L. Nakamura F. Y. Stefanello J. M. F. Pessoa L. C. V. Smirmaul B. P. C. Pereira G. (2020). Initial Validity and Reliability of the Portuguese Borg Rating of Perceived Exertion 6-20 Scale. Meas. Phys. Edu. Exerc. Sci. 24, 103–114. 10.1080/1091367X.2019.1710709

[B22] Carling C. Bloomfield J. Nelsen L. Reilly T. (2008). The Role of Motion Analysis in Elite Soccer. Sports Med. 38, 839–862. 10.2165/00007256-200838100-00004 18803436

[B23] Carling C. (2013). Interpreting Physical Performance in Professional Soccer Match-Play: Should We Be More Pragmatic in Our Approach? Sports Med. 43, 655–663. 10.1007/s40279-013-0055-8 23661303

[B24] Charness G. Gneezy U. Kuhn M. A. (2012). Experimental Methods: Between-Subject and Within-Subject Design. J. Econ. Behav. Organ. 81, 1–8. 10.1016/j.jebo.2011.08.009

[B25] Clemente F. M. Mendes B. Nikolaidis P. T. Calvete F. Carriço S. Owen A. L. (2017). Internal Training Load and its Longitudinal Relationship with Seasonal Player Wellness in Elite Professional Soccer. Physiol. Behav. 179, 262–267. 10.1016/j.physbeh.2017.06.021 28668619

[B26] Clemente F. M. Rabbani A. Araújo J. P. (2019). Ratings of Perceived Recovery and Exertion in Elite Youth Soccer Players: Interchangeability of 10-point and 100-point Scales. Physiol. Behav. 210, 112641. 10.1016/j.physbeh.2019.112641 31377310

[B27] Clemente F. M. Silva A. F. Alves A. R. Nikolaidis P. T. Ramirez-Campillo R. Lima R. (2020). Variations of Estimated Maximal Aerobic Speed in Children Soccer Players and its Associations with the Accumulated Training Load: Comparisons between Non, Low and High Responders. Physiol. Behav. 224, 113030. 10.1016/j.physbeh.2020.113030 32593751

[B28] Cobley S. Baker J. Wattie N. McKenna J. (2009). Annual Age-Grouping and Athlete Development. Sports Med. 39, 235–256. 10.2165/00007256-200939030-00005 19290678

[B29] Coppalle S. Rave G. Ben Abderrahman A. Ali A. Salhi I. Zouita S. (2019). Relationship of Pre-season Training Load with In-Season Biochemical Markers, Injuries and Performance in Professional Soccer Players. Front. Physiol. 10, 409. 10.3389/fphys.2019.00409 31031638 PMC6474299

[B30] Coutinho D. Gonçalves B. Figueira B. Abade E. Marcelino R. Sampaio J. (2015). Typical Weekly Workload of under 15, under 17, and under 19 Elite Portuguese Football Players. J. Sports Sci. 33, 1229–1237. 10.1080/02640414.2015.1022575 25789549

[B31] Coutinho D. Gonçalves B. Travassos B. Folgado H. Figueira B. Sampaio J. (2020). Different Marks in the Pitch Constraint Youth Players' Performances during Football Small-Sided Games. Res. Q. Exerc. Sport 91, 15–23. 10.1080/02701367.2019.1645938 31479411

[B32] Coutinho D. Gonçalves B. Travassos B. Wong D. P. Coutts A. J. Sampaio J. E. (2017). Mental Fatigue and Spatial References Impair Soccer Players' Physical and Tactical Performances. Front. Psychol. 8, 1645. 10.3389/fpsyg.2017.01645 28983273 PMC5613114

[B33] Coutts A. J. Crowcroft S. Kempton T. (2017). “Developing Athlete Monitoring Systems,” in Sport, Recovery, and Performance: Interdisciplinary Insights. Editor Kellmann M. (Abingdon: Routledge), 19–32. 10.4324/9781315268149-2

[B34] Cumming S. P. (2018). A Game Plan for Growth: How Football Is Leading the Way in the Consideration of Biological Maturation in Young Male Athletes. Ann. Hum. Biol. 45, 373–375. 10.1080/03014460.2018.1513560 30767617

[B35] Cumming S. P. Lloyd R. S. Oliver J. L. Eisenmann J. C. Malina R. M. (2017). Bio-banding in Sport: Applications to Competition, talent Identification, and Strength and Conditioning of Youth Athletes. Strength Cond. J. 39, 34–47. 10.1519/SSC.0000000000000281

[B36] Cumming S. P. Searle C. Hemsley J. K. Haswell F. Edwards H. Scott S. (2018). Biological Maturation, Relative Age and Self-Regulation in Male Professional Academy Soccer Players: A Test of the Underdog Hypothesis. Psychol. Sport Exerc. 39, 147–153. 10.1016/j.psychsport.2018.08.007

[B37] Duncan M. J. Roscoe C. M. P. Faghy M. Tallis J. Eyre E. L. J. (2019). Estimating Physical Activity in Children Aged 8-11 Years Using Accelerometry: Contributions from Fundamental Movement Skills and Different Accelerometer Placements. Front. Physiol. 10, 242. 10.3389/fphys.2019.00242 30936837 PMC6431656

[B38] Edwards T. Weakley J. Banyard H. G. Cripps A. Piggott B. Haff G. G. (2021). Influence of Age and Maturation Status on Sprint Acceleration Characteristics in Junior Australian Football. J. Sports Sci. 39, 1585–1593. 10.1080/02640414.2021.1886699 33583340

[B39] Ferguson C. J. (2009). An Effect Size Primer: A Guide for Clinicians and Researchers. Prof. Psychol. Res. Pract. 40, 532–538. 10.1037/a0015808

[B40] Ferraz R. Gonçalves B. Coutinho D. Marinho D. A. Sampaio J. Marques M. C. (2018b). Pacing Behaviour of Players in Team Sports: Influence of Match Status Manipulation and Task Duration Knowledge. PLoS ONE 13, e0192399. 10.1371/journal.pone.0192399 29401476 PMC5798980

[B41] Ferraz R. Gonçalves B. Coutinho D. Oliveira R. Travassos B. Sampaio J. (2020). Effects of Knowing the Task's Duration on Soccer Players' Positioning and Pacing Behaviour during Small-Sided Games. Ijerph 17, 3843. 10.3390/ijerph17113843 PMC7312572 32481705

[B42] Ferraz R. Gonçalves B. Van Den Tillaar R. Jiménez Sáiz S. Sampaio J. Marques M. C. (2018a). Effects of Knowing the Task Duration on Players' Pacing Patterns during Soccer Small-Sided Games. J. Sports Sci. 36, 116–122. 10.1080/24733938.2017.1283433 28134013

[B43] Figueiredo A. J. Coelho-e-Silva M. J. Cumming S. P. Malina R. M. (2019). Relative Age Effect: Characteristics of Youth Soccer Players by Birth Quarter and Subsequent Playing Status. J. Sports Sci. 37, 677–684. 10.1080/02640414.2018.1522703 30246606

[B44] Figueiredo P. Seabra A. Brito M. Galvão M. Brito J. (2021). Are Soccer and Futsal Affected by the Relative Age Effect? the Portuguese Football Association Case. Front. Psychol. 12, 679476. 10.3389/fpsyg.2021.679476 34122274 PMC8194498

[B110] Flanagan E. P. (2013). The Effect Size Statistic—Applications for the Strength and Conditioning Coach. Strength Cond. J. 35 (5), 37–40. 10.1519/SSC.0b013e3182a64d20

[B45] Folgado H. Gonçalves B. Sampaio J. (2018). Positional Synchronization Affects Physical and Physiological Responses to Preseason in Professional Football (Soccer). Res. Sports Med. 26, 51–63. 10.1080/15438627.2017.1393754 29058465

[B46] Gabbett T. J. Hulin B. Blanch P. Chapman P. Bailey D. (2019). To Couple or Not to Couple? for Acute:chronic Workload Ratios and Injury Risk, Does it Really Matter? Int. J. Sports Med. 40, 597–600. 10.1055/a-0955-5589 31291651

[B47] Gallo T. F. Cormack S. J. Gabbett T. J. Lorenzen C. H. (2016). Pre-training Perceived Wellness Impacts Training Output in Australian Football Players. J. Sports Sci. 34, 1445–1451. 10.1080/02640414.2015.1119295 26637525

[B48] Gómez-Carmona C. D. Bastida-Castillo A. Ibáñez S. J. Pino-Ortega J. (2020). Accelerometry as a Method for External Workload Monitoring in Invasion Team Sports. A Systematic Review. PLoS ONE 15, e0236643. 10.1371/journal.pone.0236643 32841239 PMC7447012

[B49] Gonçalves B. Coutinho D. Exel J. Travassos B. Lago C. Sampaio J. (2019). Extracting Spatial-Temporal Features that Describe a Team Match Demands when Considering the Effects of the Quality of Opposition in Elite Football. PLoS ONE 14, e0221368. 10.1371/journal.pone.0221368 31437220 PMC6705862

[B50] Gonçalves B. Coutinho D. Travassos B. Folgado H. Caixinha P. Sampaio J. (2018). Speed Synchronization, Physical Workload and Match-To-Match Performance Variation of Elite Football Players. PLoS ONE 13, e0200019. 10.1371/journal.pone.0200019 30040849 PMC6057640

[B51] Haddad M. Stylianides G. Djaoui L. Dellal A. Chamari K. (2017). Session-RPE Method for Training Load Monitoring: Validity, Ecological Usefulness, and Influencing Factors. Front. Neurosci. 11, 612. 10.3389/fnins.2017.00612 29163016 PMC5673663

[B52] Hill M. Scott S. Malina R. M. McGee D. Cumming S. P. (2020). Relative Age and Maturation Selection Biases in Academy Football. J. Sports Sci. 38, 1359–1367. 10.1080/02640414.2019.1649524 31366286

[B53] Hill M. Scott S. McGee D. Cumming S. P. (2021). Are Relative Age and Biological Ages Associated with Coaches' Evaluations of Match Performance in Male Academy Soccer Players? Int. J. Sports Sci. Coaching 16, 227–235. 10.1177/1747954120966886

[B54] Hopkins W. G. Marshall S. W. Batterham A. M. Hanin J. (2009). Progressive Statistics for Studies in Sports Medicine and Exercise Science. Med. Sci. Sports Exerc. 41, 3–12. 10.1249/MSS.0b013e31818cb278 19092709

[B55] Hung C.-H. Clemente F. M. Bezerra P. Chiu Y.-W. Chien C.-H. Crowley-McHattan Z. (2020). Post-exercise Recovery of Ultra-short-term Heart Rate Variability after Yo-Yo Intermittent Recovery Test and Repeated Sprint Ability Test. Ijerph 17, 4070. 10.3390/ijerph17114070 PMC7312126 32517382

[B56] Impellizzeri F. M. Marcora S. M. Coutts A. J. (2019). Internal and External Training Load: 15 Years on. Int. J. Sports Physiol. Perform. 14, 270–273. 10.1123/ijspp.2018-0935 30614348

[B57] Impellizzeri F. M. Rampinini E. Marcora S. M. (2005). Physiological Assessment of Aerobic Training in Soccer. J. Sports Sci. 23, 583–592. 10.1080/02640410400021278 16195007

[B58] Issurin V. B. (2010). New Horizons for the Methodology and Physiology of Training Periodization. Sports Med. 40, 189–206. 10.2165/11319770-000000000-00000 20199119

[B59] Johnson A. Farooq A. Whiteley R. (2017). Skeletal Maturation Status Is More Strongly Associated with Academy Selection Than Birth Quarter. Sci. Med. Football 1, 157–163. 10.1080/24733938.2017.1283434

[B60] Kelly A. Wilson M. R. Jackson D. T. Goldman D. E. Turnnidge J. Côté J. (2021). A Multidisciplinary Investigation into "Playing-Up" in Academy Football According to Age Phase. J. Sports Sci. 39, 854–864. 10.1080/02640414.2020.1848117 33203302

[B61] Kenttä G. Hassmén P. (1998). Overtraining and Recovery. Sports Med. 26, 1–16. 10.2165/00007256-199826010-00001 9739537

[B62] Koo T. K. Li M. Y. (2016). A Guideline of Selecting and Reporting Intraclass Correlation Coefficients for Reliability Research. J. Chiropractic Med. 15, 155–163. 10.1016/j.jcm.2016.02.012 PMC4913118 27330520

[B63] Linke D. Link D. Lames M. (2018). Validation of Electronic Performance and Tracking Systems EPTS under Field Conditions. PLoS ONE 13, e0199519. 10.1371/journal.pone.0199519 30036364 PMC6056042

[B64] Lloyd R. S. Oliver J. L. (2012). The Youth Physical Development Model. Strength Cond. J. 34, 61–72. 10.1519/SSC.0b013e31825760ea

[B65] Lovell R. Fransen J. Ryan R. Massard T. Cross R. Eggers T. (2019). Biological Maturation and Match Running Performance: A National Football (Soccer) Federation Perspective. J. Sci. Med. Sport 22, 1139–1145. 10.1016/j.jsams.2019.04.007 31056279

[B66] Lupo C. Boccia G. Ungureanu A. N. Frati R. Marocco R. Brustio P. R. (2019a). The Beginning of Senior Career in Team Sport Is Affected by Relative Age Effect. Front. Psychol. 10, 1465. 10.3389/fpsyg.2019.01465 31293489 PMC6606777

[B67] Lupo C. Capranica L. Cortis C. Guidotti F. Bianco A. Tessitore A. (2017). Session-RPE for Quantifying Load of Different Youth Taekwondo Training Sessions. J. Sports Med. Phys. Fitness 57, 189–194. 10.23736/s0022-4707.16.06021-x 26796074

[B68] Lupo C. Ungureanu A. N. Frati R. Panichi M. Grillo S. Brustio P. R. (2020b). Player Session Rating of Perceived Exertion: A More Valid Tool Than Coaches' Ratings to Monitor Internal Training Load in Elite Youth Female Basketball. Int. J. Sports Physiol. Perform. 15, 548–553. 10.1123/ijspp.2019-0248 31693998

[B69] Malcata R. M. Hopkins W. G. (2014). Variability of Competitive Performance of Elite Athletes: A Systematic Review. Sports Med. 44, 1763–1774. 10.1007/s40279-014-0239-x 25108349

[B70] Malina R. M. Cumming S. P. Rogol A. D. Coelho-e-Silva M. J. Figueiredo A. J. Konarski J. M. (2019). Bio-banding in Youth Sports: Background, Concept, and Application. Sports Med. 49, 1671–1685. 10.1007/s40279-019-01166-x 31429034

[B71] Malina R. M. Rogol A. D. Cumming S. P. Coelho e Silva M. J. Figueiredo A. J. (2015). Biological Maturation of Youth Athletes: Assessment and Implications. Br. J. Sports Med. 49, 852–859. 10.1136/bjsports-2015-094623 26084525

[B72] Maughan P. C. MacFarlane N. G. Swinton P. A. (2021). Quantification of Training and Match-Play Load across a Season in Professional Youth Football Players. Int. J. Sports Sci. Coaching 16, 1169–1177. 10.1177/17479541211000328

[B73] Miguel M. Oliveira R. Loureiro N. García-Rubio J. Ibáñez S. J. (2021). Load Measures in Training/match Monitoring in Soccer: A Systematic Review. Ijerph 18, 2721. 10.3390/ijerph18052721 33800275 PMC7967450

[B74] Mirwald R. L. G. Baxter-jones A. D. Bailey D. A. Beunen G. P. (2002). An Assessment of Maturity from Anthropometric Measurements. Med. Sci. Sports Exerc. 34, 689–694. 10.1097/00005768-200204000-0002010.1249/00005768-200204000-00020 11932580

[B75] Nikolaidis P. T. Clemente F. M. van der Linden C. M. I. Rosemann T. Knechtle B. (2018). Validity and Reliability of 10-Hz Global Positioning System to Assess In-Line Movement and Change of Direction. Front. Physiol. 9, 1–7. 10.3389/fphys.2018.00228 29599725 PMC5862865

[B76] Nobari H. Alves A. R. Clemente F. M. Pérez-Gómez J. Clark C. C. T. Granacher U. (2021b). Associations between Variations in Accumulated Workload and Physiological Variables in Young Male Soccer Players over the Course of a Season. Front. Physiol. 12, 638180. 10.3389/fphys.2021.638180 33815144 PMC8012769

[B77] Nobari H. Alves A. R. Haghighi H. Clemente F. M. Carlos-Vivas J. Pérez-Gómez J. (2021c). Association between Training Load and Well-Being Measures in Young Soccer Players during a Season. Ijerph 18, 4451. 10.3390/ijerph18094451 33922250 PMC8122726

[B78] Nobari H. Silva A. F. Clemente F. M. Siahkouhian M. García-Gordillo M. Á. Adsuar J. C. (2021a). Analysis of Fitness Status Variations of Under-16 Soccer Players over a Season and Their Relationships with Maturational Status and Training Load. Front. Physiol. 11, 1840. 10.3389/fphys.2020.597697 PMC7892949 33613301

[B79] O’Donoghue P. (2004). Sources of Variability in Time-Motion Data; Measurement Error and within Player Variability in Work-Rate. Int. J. Perform. Anal. Sport 4, 42–49. 10.1080/24748668.2004.11868303

[B80] Oliveira R. Brito J. P. Moreno-Villanueva A. Nalha M. Rico-González M. Clemente F. M. (2021). Reference Values for External and Internal Training Intensity Monitoring in Young Male Soccer Players: A Systematic Review. Healthcare 9, 1567. 10.3390/healthcare9111567 34828613 PMC8622615

[B81] Otte F. W. Millar S.-K. Klatt S. (2019). Skill Training Periodization in "Specialist" Sports Coaching-An Introduction of the "PoST" Framework for Skill Development. Front. Sports Act. Living 1, 61. 10.3389/fspor.2019.00061 33344984 PMC7739686

[B82] Patel R. Nevill A. Cloak R. Smith T. Wyon M. (2019). Relative Age, Maturation, Anthropometry and Physical Performance Characteristics of Players within an Elite Youth Football Academy. Int. J. Sports Sci. Coaching 14, 714–725. 10.1177/1747954119879348

[B83] Patel R. Nevill A. Smith T. Cloak R. Wyon M. (2020). The Influence of Birth Quartile, Maturation, Anthropometry and Physical Performances on Player Retention: Observations from an Elite Football Academy. Int. J. Sports Sci. Coaching 15, 121–134. 10.1177/1747954120906507

[B84] Rago V. Brito J. Figueiredo P. Krustrup P. Rebelo A. (2020). Application of Individualized Speed Zones to Quantify External Training Load in Professional Soccer. J. Hum. Kinet. 72, 279–289. 10.2478/hukin-2019-0113 32269668 PMC7126260

[B85] Rojas-Valverde D. Gómez-Carmona C. D. Gutiérrez-Vargas R. Pino-Ortega J. (2019). From Big Data Mining to Technical Sport Reports: the Case of Inertial Measurement Units. BMJ Open Sport Exerc. Med. 5, e000565–3. 10.1136/bmjsem-2019-000565 PMC6797247 31673403

[B86] Rojas-Valverde D. Pino-Ortega J. Gómez-Carmona C. D. Rico-González M. (2020). A Systematic Review of Methods and Criteria Standard Proposal for the Use of Principal Component Analysis in Team's Sports Science. Ijerph 17, 8712. 10.3390/ijerph17238712 PMC7727687 33255212

[B87] Rubajczyk K. Rokita A. (2018). The Relative Age Effect in Poland's Elite Youth Soccer Players. J. Hum. Kinet. 64, 265–273. 10.1515/hukin-2017-0200 30429917 PMC6231332

[B88] Ryan D. Lewin C. Forsythe S. McCall A. (2018). Developing World-Class Soccer Players: An Example of the Academy Physical Development Program from an English Premier League Team. Strength Cond J. 40, 2–11. 10.1519/SSC.0000000000000340

[B89] Saavedra-García M. Matabuena M. Montero-Seoane A. Fernández-Romero J. J. (2019). A New Approach to Study the Relative Age Effect with the Use of Additive Logistic Regression Models: A Case of Study of FIFA Football Tournaments (1908-2012). PLoS ONE 14, e0219757. 10.1371/journal.pone.0219757 31310610 PMC6634404

[B90] Salter J. De Ste Croix M. B. A. Hughes J. D. Weston M. Towlson C. (2021). Monitoring Practices of Training Load and Biological Maturity in UK Soccer Academies. Int. J. Sports Physiol. Perform. 16, 395–406. 10.1123/ijspp.2019-0624 33401237

[B91] Santos F. J. Caldeira Ferreira C. Figueiredo T. P. Espada M. C. (2021). Influence of Different 1v1 Small-Sided Game Conditions in Internal and External Load of U-15 and U-12 Soccer Players. Trends Sport Sci. 29, 45–53.

[B92] Sarmento H. Anguera M. T. Pereira A. Araújo D. (2018a). Talent Identification and Development in Male Football: A Systematic Review. Sports Med. 48, 907–931. 10.1007/s40279-017-0851-7 29299878

[B93] Sarmento H. Clemente F. M. Araújo D. Davids K. McRobert A. Figueiredo A. (2018b). What Performance Analysts Need to Know about Research Trends in Association Football (2012-2016): A Systematic Review. Sports Med. 48, 799–836. 10.1007/s40279-017-0836-6 29243038

[B94] Simmons C. Paull G. C. (2001). Season-of-birth Bias in Association Football. J. Sports Sci. 19, 677–686. 10.1080/02640410152475801 11522143

[B95] Skorski S. Skorski S. Faude O. Hammes D. Meyer T. (2016). The Relative Age Effect in Elite German Youth Soccer: Implications for a Successful Career. Int. J. Sports Physiol. Perform. 11, 370–376. 10.1123/ijspp.2015-0071 26308489

[B96] Teixeira J. E. Forte P. Ferraz R. Leal M. Ribeiro J. Silva A. J. (2021a). Monitoring Accumulated Training and Match Load in Football: A Systematic Review. Ijerph 18, 3906. 10.3390/ijerph18083906 33917802 PMC8068156

[B97] Teixeira J. E. Forte P. Ferraz R. Leal M. Ribeiro J. Silva A. J. (2021b). Quantifying Sub-elite Youth Football Weekly Training Load and Recovery Variation. Appl. Sci. 11, 4871. 10.3390/app11114871

[B98] Teixeira J. E. Leal M. Ferraz R. Ribeiro J. Cachada J. M. Barbosa T. M. (2021c). Effects of Match Location, Quality of Opposition and Match Outcome on Match Running Performance in a Portuguese Professional Football Team. Entropy 23, 973. 10.3390/e23080973 34441113 PMC8391710

[B99] Towlson C. Cobley S. Midgley A. Garrett A. Parkin G. Lovell R. (2017). Relative Age, Maturation and Physical Biases on Position Allocation in Elite-Youth Soccer. Int. J. Sports Med. 38, 201–209. 10.1055/s-0042-119029 28219108

[B100] Trecroci A. Boccolini G. Duca M. Formenti D. Alberti G. (2020a). Mental Fatigue Impairs Physical Activity, Technical and Decision-Making Performance during Small-Sided Games. PLoS ONE 15, e0238461. 10.1371/journal.pone.0238461 32903263 PMC7480836

[B101] Trecroci A. Porcelli S. Perri E. Pedrali M. Rasica L. Alberti G. (2020b). Effects of Different Training Interventions on the Recovery of Physical and Neuromuscular Performance after a Soccer Match. J. Strength Cond. Res. 34, 2189–2196. 10.1519/JSC.0000000000003269 31373975

[B102] Vanrenterghem J. Nedergaard N. J. Robinson M. A. Drust B. (2017). Training Load Monitoring in Team Sports: A Novel Framework Separating Physiological and Biomechanical Load-Adaptation Pathways. Sports Med. 47, 2135–2142. 10.1007/s40279-017-0714-2 28283992

[B103] Whitehead S. Till K. Weaving D. Jones B. (2018). The Use of Microtechnology to Quantify the Peak Match Demands of the Football Codes: A Systematic Review. Sports Med. 48, 2549–2575. 10.1007/s40279-018-0965-6 30088218 PMC6182461

[B104] Williams S. West S. Cross M. J. Stokes K. A. (2017). Better Way to Determine the Acute:chronic Workload Ratio? Br. J. Sports Med. 51, 209–210. 10.1136/bjsports-2016-096589 27650255

[B105] Wrigley R. Drust B. Stratton G. Scott M. Gregson W. (2012). Quantification of the Typical Weekly In-Season Training Load in Elite Junior Soccer Players. J. Sports Sci. 30, 1573–1580. 10.1080/02640414.2012.709265 22852843

[B106] Yi Q. Gómez M.-Á. Liu H. Gao B. Wunderlich F. Memmert D. (2020a). Situational and Positional Effects on the Technical Variation of Players in the UEFA Champions League. Front. Psychol. 11, 1201. 10.3389/fpsyg.2020.01201 32636779 PMC7318796

[B107] Yi Q. Gómez-Ruano M.-Á. Liu H. Zhang S. Gao B. Wunderlich F. (2020b). Evaluation of the Technical Performance of Football Players in the UEFA Champions League. Ijerph 17, 604. 10.3390/ijerph17020604 PMC7013673 31963565

[B108] Younesi S. Rabbani A. Manuel Clemente F. Sarmento H. Figueiredo A. (2021). Session-to-session Variations of Internal Load during Different Small-Sided Games: A Study in Professional Soccer Players. Res. Sports Med. 29, 462–474. 10.1080/15438627.2021.1888103 33573422

[B109] Zurutuza U. Castellano J. Echeazarra I. Casamichana D. (2017). Absolute and Relative Training Load and its Relation to Fatigue in Football. Front. Psychol. 8, 878. 10.3389/fpsyg.2017.00878 28634456 PMC5459919

